# Budget impact analysis of a multifaceted nurse-led intervention to reduce indwelling urinary catheter use in New South Wales Hospitals

**DOI:** 10.1186/s12913-022-08313-7

**Published:** 2022-08-05

**Authors:** Rod Ling, Michelle Giles, Andrew Searles

**Affiliations:** 1 Hunter Medical Research Institute, Lot 1, Kookaburra Circuit, New Lambton Heights, NSW 2305 Australia; 2 University of Newcastle, School of Medicine and Public Health, Callaghan, NSW 2308 Australia; 3 Hunter New England Local Health District, Nursing and Midwifery Centre, Gate Cottage James Fletcher Campus, 72 Watt Street, Newcastle, NSW 2300 Australia

**Keywords:** Budget Impact Analysis, Catheters, Nurses, Urinary Tract Infections

## Abstract

**Background:**

In hospitals, catheter acquired urinary tract infection causes significant resource waste and discomfort among admitted patients. An intervention for reducing indwelling catheterisations - No-CAUTI - was trialled across four hospitals in New South Wales, Australia. No-CAUTI includes: train-the-trainer workshops, site champions, compliance audits, and point prevalence surveys. The trial showed reductions on usual care catheterisation rates at 4- and 9-month post-intervention. This result was statistically non-significant; and post-intervention catheterisation rates rebounded between 4 and 9 months. However, No-CAUTI showed statistically significant catheterisation decreases for medical wards, female patients and for short-term catheterisations. This study presents a budget impact analysis of a projected five year No-CAUTI roll out across New South Wales public hospitals, from the cost perspective of the New South Wales Ministry of Health.

**Methods:**

Budget forecasts were made for five year roll outs of: i) No-CAUTI; and ii) usual care, among all public hospitals in New South Wales hosting overnight stays (n=180). The roll out design maintains intervention effectiveness with ongoing workshops, quality audits, and hospital surveys. Forecasts of catheterisations, procedures and treatments were modelled on No-CAUTI trial observations. Costs were sourced from trial records, the Medical Benefits Scheme, the Pharmaceutical Benefits Scheme and public wage awards. Cost and parameter uncertainties were considered with sensitivity scenarios.

**Results:**

The estimated five-year No-CAUTI roll-out cost was $1.5 million. It had an overall budget saving of $640,000 due to reductions of 100,100 catheterisations, 33,300 urine tests and 6,700 antibiotics administrations. Non-Metropolitan hospitals had a net saving of $1.2 million, while Metropolitan hospitals had a net cost of $0.54 million.

**Conclusions:**

Compared to usual care, NO-CAUTI is expected to realise overall budget savings and decreases in catheterisations over five years. These findings allow a consideration of the affordability of a wide implementation.

**Trial registration:**

Registered with the Australian New Zealand Clinical Trials Registry (ACTRN12617000090314). First registered 17 January 2017, retrospectively. First enrolment, 15/11/2016.

**Supplementary Information:**

The online version contains supplementary material available at 10.1186/s12913-022-08313-7.

## Introduction

Waste in healthcare is a significant problem. It is evidenced by unexplained variations in: the prices of interventions and procedures [1]; the overuse, underuse and misuse of health technologies [2]; technologies that deliver little or no health benefit to patients; variations in safety and quality; and the provision of non-guideline care [2–5]. While the extent of waste in the healthcare system is difficult to specify, there are published estimates. The Productivity Commission has estimated that the efficiency of Australian healthcare could be improved by approximately 20% by using evaluation to make better decisions on what healthcare is implemented and what is removed [3].

Catheter-associated urinary tract infections (CAUTIs) make up 70 - 80% of all hospital-associated urinary tract infections (HAUTIs) [6, 7]. Also, according to Mitchell et al [8], there are approximately 380,600 extra public hospital bed-days used each year in Australia due to HAUTIs (the majority being catheter-associated). An effective approach to lowering CAUTI has been to decrease catheter insertion rates combined with an increase in the rate of timely catheter removal [9]. CAUTI ‘bundles’ - a collection of evidence-based practices directed toward an improvement in CAUTI rates - are a common improvement strategy [9]. An example is the ‘bladder bundle’, for which there is a nurse-initiated catheter removal protocol, catheter removal reminders, ultrasound bladder monitoring, and catheter insertion maintenance and care [10]. CAUTI can be viewed as a ‘nurse-sensitive indicator’, that is, as a condition with a critical positive association with levels of nursing care [11]. Therefore, reducing CAUTI and catheter use requires the involvement of nursing staff, and nurse-led interventions with demonstrated effectiveness in reducing urinary catheter usage rates and CAUTI [12].

A trial of such an intervention was conducted over four hospitals in the state of New South Wales (NSW), Australia, between 2017 and 2018: the ‘No-CAUTI’ trial. The intervention aimed to optimise the use of a health technology (indwelling urinary catheters (IDC)) in hospital settings. This intervention has the potential to reduce unnecessary urinary catheterisation procedures and also increase adherence to evidence-based guideline care when urinary catheterisations are warranted. Both can potentially reduce CAUTI, along with the associated and consequential CAUTI-related treatment costs [13–16].

Economic evaluations (e.g. cost effectiveness analysis) of such interventions and their alternatives are often required as an input to healthcare decision-making. These analyses are typically designed to reflect the relative *efficiency* of various courses of action that decision makers could choose. However, there is growing evidence that these analyses are not well understood by healthcare decision makers so they may not be optimally contributing to decision-making [5]. Also, economic analyses do not necessarily inform whether investment in the technology is affordable. That is, a health technology might be cost-effective but not affordable for the implementing healthcare service [5].

Budget impact analysis (BIA) supports healthcare decisions by informing the relative ‘affordability’ of intervention choices by comparing expected budgets for alternative proposals; both in terms of cash outlays and cash savings. BIA also compares intervention outcomes for alternative services [17]. Unlike cost effectiveness analysis, which gives ratios of relative efficiency in terms of opportunity costs, BIA focuses on the comparative budget costs (or financial costs) of implementing a new intervention in comparison to those of usual care or an alternative intervention. The standard audiences for BIAs are health service decision makers [18, 19] for whom BIAs provide useful information for assessments of affordability of proposals [20]. Specialist literature, published largely by the Professional Society for Health Economics and Outcomes Research (ISPOR) [17, 21, 22] sets out well accepted aims and best practices for BIA. Several countries, influenced by, or associated with ISPOR, have also published their own official BIA guidelines [18, 23, 24].

To the author’s best knowledge, no previous budget impact analyses have been published on nurse led hospital interventions for reducing catheterisation incidence (Additional file 1: Literature Review). While studies of similar interventions employing financial costings have been published [25, 26], none has produced a detailed impact statement of projected budget costs and savings for decision makers. Economic studies of hospital CAUTI reduction programs, that have attempted to improve and standardise catheterisation practice have shown effectiveness, but usually at a positive economic cost [27] or with negative incremental costs [28–30]. As a novel intervention specific to nursing practice and focused on reducing CAUTI through catheterisation reduction, the No-CAUTI intervention warrants further consideration with budget impact analysis.

## Methods

### Aim

The aim of this study is to forecast and compare budget costs and outcomes across New South Wales public hospitals for roll outs of: a) No-CAUTI; and b) usual care, both over five years. The costing perspective is that of the New South Wales Ministry of Health [31].

### Source data for the BIA: the No-CAUTI trial

The No-CAUTI trial researched a nurse led multi-faceted intervention conducted in the Australian state of New South Wales (NSW) in 2017-18 [13, 14, 16, 32]. The primary objective of the trial was to decrease catheterisation rates to reduce the risk of CAUTI [16]. A standardised generic catheter insertion pack with catheterisation equipment, documentation stickers, and securing devices, was developed, and distributed across four hospitals, in two NSW local health districts (LHDs) - Central Coast LHD and Hunter New England LHD. The intervention team developed electronic data collection tools to support point prevalence and compliance auditing processes. The research design was a cluster-controlled pre-post study at a facility level with a phased intervention implementation approach [13]. Three comprehensive point prevalence patient surveys, collecting catheterisation, and treatment data were conducted: pre-intervention, post-intervention 4 months and post intervention 9 months. Two hospitals are in metropolitan locations, while two are regional/rural facilities. Separately, the metropolitan hospitals have bed capacities of 550 and 318; and each has an intensive care unit (ICU)s. The rural hospitals have respective capacities of 360 and 260 beds.

In both LHDs, the intervention required six months for implementation, comprising: three hour ‘Train-the-Trainer’ workshops and ward in-services (over three to four weeks)), which were expected to generate ongoing on-site education in future years; champion meetings for six months post education; compliance audits; and promotion (posters, badges etc). Further details can be found elsewhere [16].

As noted eslsewhere [13], the No-CAUTI trial showed a non-significant trend towards reduction of catheter prevalence across the three timepoints (11.96%, 9.90%, 10.19%). However, the authors hold that consideration of No-CAUTI for roll-out is warranted for two reasons.

First, given catheterisation rates pre-intervention (12%) were lower than rates reported in the literature (15% to 25%) [2, 3], the 2% overall reduction in catheter prevalence to 10% post-intervention is clinically significant. Notably, reductions in catheter prevalence were more marked in hospitals that started with a higher baseline, evidenced by a statistically significant improvement in urinary catheterisations at one rural hospital from pre-intervention to 9-months post-intervention (16% to 8%, *p* < .01) [13].

Second, statistically significant catheter prevalence reductions between pre-intervention and 9-months post-intervention occurred among important sub-groups. Notably this occurred for female patients (11% - 8%; *p* = .015), but this result was offset by no change among male patients (13% - 13%). The largest specialty group, those in medical wards, showed a statistically significant drop in catheter prevalence from pre-intervention to 4 months intervention (9% to 6%, *p* = .02) [13].

For the same period, a statistically significant downward change also occurred for patients receiving short-term catheterisations (≤ 3 days) (*p* = .023). Further, catheterisations among patients requiring postsurgical urinary management - which are avoidable – decreased between pre-intervention to 9-month post intervention from 29%–21%. Hence, effectiveness can be confidently predicted for certain major groups in a roll out, albeit with efforts required to gain improvements among others [13].

### Budget impact analysis

#### Budget perspective

The costing perspective for any BIA is that of the decision maker – individual or organisation - responsible for managing the program budget of an intervention, – usually referred to as the ‘budget holder’ [17, 19, 21–24, 33]. Hence, this BIA has the perspective of the NSW Ministry of Health, which is responsible for funding NSW public hospitals [31, 34]. All costs were collected at the time of the No-CAUTI implementation (2017-18) and were inflation adjusted to AUD 2019-20 costs with indexes published by the Australian Institute of Health and Welfare [35].

#### Study population

The study population includes the estimated overnight separations of NSW public hospitals for five annual operating periods [36]. Selected hospitals were from seven classes: principal referral (n=11), women’s (n=1), public acute groups A (n=23), B (n=17), C (n=40) and D (n=60) and ‘very small’ (n=28) (Additional file 1: NSW Hospital Summary) [36]. Annual overnight separations for a five year time horizon, were estimated with linear forecasts based on published data from successive New South Wales Ministry Annual Reports (Additional file 1: NSW Overnight Stays Forecast) [37].

#### Time horizon

The budget impact analysis provides annual budget forecasts of NSW No-CAUTI implementation for five years (2022-23 to 2026-27). This duration was advised by author MG, a senior co-ordinator of the No-CAUTI trial, as appropriate for planning purposes. As with ISPOR practice, no discounting was undertaken [20].

#### Treatment mix

This study will analyse and compare five year budget costs for two mutually exclusive scenarios involving the study population for: a) usual care; and b) the No-CAUTI intervention

#### Outcomes

The main outcome of this BIA exercise is the net cost/saving attributable to the No-CAUTI intervention over the time horizon. Other important outcomes are, the direct budget cost of the No-CAUTI intervention; scenario differences in catheterisations, urine tests and antibiotic administrations.

#### Modelling and presentation

A BIA model was constructed in a Microsoft EXCEL 2016 [38] workbook. Results are presented to show forecast intervention impacts - catheterisations, urine tests, antibiotic administrations - and their costs, comparatively to usual care. This will inform decision makers how No-CAUTI will impact on their operational budgets.

#### No-CAUTI intervention roll-out program

The basic method for designing the state-wide roll-out had two parts. First, using hospital data, estimations were made of resources necessary for roll-out of three critical aspects of the No-CAUTI intervention: workshops, compliance auditing and point prevalence surveying (Additional file 1: Workshops; Audits Point Prev) [34, 39]. In Year 1, there would be 144 online workshops across all 180 hospitals - larger hospitals having multiple workshops and very small hospitals participating in multi-hospital sessions.

Note that as the trial showed an increase in the catheterisation rate between 4- and 9-months post intervention, this implies a decay in effect size by 9-months post intervention. To counter effect dilution, from Year 2 there will be 16 continuing workshops (one for each LHD) which may be attended by new trainer nurses or qualified No-CAUTI trainers seeking refresher instruction. Intervention staff will continuing liaison with hospitals to encourage maintenance of No-CAUTI practices.

Also, in Year 1, each hospital will conduct five compliance audits and two catheterisation point prevalence surveys. In subsequent years there will be one audit and one point prevalence survey. Results will be reported to the program managers and presented to the Ministry of Health.

For the second part of the roll out design, a program management scenario was created by estimating labour hours and resource costs required for each aspect. Time is budgeted to allow program staff to monitor progress among hospitals, supporting program effectiveness *(*Additional file 1: Labour_Time_Budget) (Table 1).

**Table 1:** Intervention budget costs years 1 to 5

	**Items**	**Totals**
	**($)**	($)
**YEAR 1**		
**Labour**		
2 * Co-ordinator: (CNC3) @ 0.7 FTE	$172,851	
1* Administrator (ACO6) @ 1 FTE	$94,327	
* Add: Labour On-Costs (21.5%)*	$57,470	
**Total Labour Costs**		**$324,648 **
**Other Costs**		
Overheads (27.5% of labour cost)	$73,474	
Contingency Allocation	$10,000	
**Total Other Costs**		**$83,474 **
**TOTAL YEAR 1 **		**$408,122 **
**YEAR 2**		
**Labour**		
1 * Co-ordinator: (CNC3) @ 0.8 FTE	$98,772	
1 * Administrator (ACO6) @ 0.8 FTE	$75,462	
* Add: Labour On-Costs (21.5%)*	$37,478	
**Total Labour Costs**		**$211,711 **
**Other Costs**		
Overheads (27.5% of labour cost)	$47,914	
Contingency Expenses	$10,000	
**Total Other Costs**		**$57,914 **
**TOTAL YEAR 2**		**$269,625 **
**YEARS 3 to 5 **		
TOTAL YEARS 3 TO 5 (Year 2 Total * 3)		**$808,876 **
**TOTAL YEARS 1 TO 5**		**$1,486,623 **

#### Estimates of resource use

Workshops will be conducted remotely by intervention staff on electronic media. The number of workshops required per hospital was estimated on an algorithm related to bed capacities shown in Additional file 1: Workshops; Audits Point-Prev*.* The trial also included No-CAUTI promotional materials (laminated posters, flyers, and badges). For the roll out, these materials - already developed in the trial - will exclude badges. This will leave posters and flyers to be distributed by email for printing at the sites. The intervention costs of distributing the materials include the labour time for the administrator to telephone each hospital to confirm that materials have been sent. All annual compliance audits and point prevalence surveys will be conducted by permanent hospital staff. Hence audits and surveys will not require extra budget costs.

#### Intervention budget costs

Intervention labour time was budgeted *(*Additional file 1: Labour Time Budget) and costed (Table 1) in 2019-2020 Australian dollars [35]. In Year 1, the program should be staffed by two Clinical Nurse Consultants grade 3 (CNC3) project co-ordinator/managers employed at 0.7 FTE. They will be supported by an Administrative and Clerical Officer Grade 6 (ACO6) working at 1.0 FTE. The nurses will conduct all workshop training by remote electronic delivery, covering training cost with their salaries. Further, the staff will spend time liaising with hospitals, making various reports to the Ministry of Health (e.g. practice audits and point prevalence results). Combined labour costs will be $172,851 [40] for the CNC3s; and $94,327 [41] for the administrative officer (Table 1).

Labour on-costs (payroll tax, superannuation etc) and associated overheads (electricity, water etc) are valued with respect to total labour costs respectively at 21.5% and 27.5%. The on-cost rate was estimated from information at a university website [42]. The overhead rate is based on personal correspondence from a finance officer at the John Hunter Hospital, Newcastle, NSW (personal email to author RL, 13/12/2018). Allowing for an extra $10,000 for contingencies, total intervention costs in Year 1 would be $408,122 (Table 1).

From Year 2, the number of workshops would be reduced due to the need to only teach new train-the-trainers or others requiring refresher sessions. Hence, the time budget allows for one annual workshop per local health district (LHD) (n=16). Such a task, along with hospital and health authority liaison could be covered by one CNC3 nurse and the administrative officer. Both could be effectively hired at 0.8 FTE. (Additional file 1: Labour Time Budget) Reduced annual program costs for Years 2 to 5 are estimated at $269,625. The total cost of the program over five years is estimated at $1,486,623 (Table 1).

Note that some No-CAUTI trial activities were not costed. For example, ward-in-services are part of usual practice and would require no changes to budgets. Champion meetings were also not costed as they would not be part of rollout (as advised by author, MG).

#### Model parameters estimations

For its parameters, this budget impact analysis requires estimates of annual rates for hospital catheterisations, urine tests and antibiotic administration for a) one full year post-intervention care; and b) one full year of pre-intervention usual care. The trial data offered only point prevalence parameter estimates, necessitating rate modelling.

Analysis rates for a 12-month post intervention period, were modelled with polynomial regressions using trial point prevalence rates as inputs. Rates were generated for catheterisations, urine testing and antibiotic administrations. The modelling can be viewed in Additional file 1: Model NSW.

For a year of usual care, the No-CAUTI trial results offered only one set of rates – those collected for ‘pre-intervention’. Hence, in the absence of more data, this analysis will forecast the usual care budget with No-CAUTI trial pre-intervention rates for catheterisations, urine culture testing and antibiotics.

All parameter rates were weighted to allow for the distribution of Metropolitan and Non-Metropolitan hospitals in NSW, which differed slightly to that of those within the trial (Additional file 1: Model Weights) (Table 2).

**Table 2:** Base case budget costs, parameters, treatment units

**1. Costs**		
**a. Catheterisations**	**($)**	
Catheterisations (Catheter Packs)^a^	$8.14	
**b. Urinary Infection**		
Urine Tests (pre CAUTI)^b^	$35.23	
Antibiotic Administrations (pre and post CAUTI)^c^	$19.48	
**2. Estimated Annual Parameter Rates**^**d**^		
	**Pre-Intervention**^**e**^	**Post-Intervention**^**f**^
	**(%)**	**(%)**
Catheterisations (per patient)	11.97%	10.27%
Urine Tests (Per catheterised patient)	43.46%	45.19%
Antibiotic Administrations (per catheterised patient)	7.60%	7.76%

^a^No-CAUTI Research records

^b^MBS Items 69333 and 73930

^c^Based on pooled antibiotic data from Pharmaceutical Benefits Scheme

^d^Weighted for distribution differences between Metropolitan and Non-Metropolitan hospitals for No-CAUTI Trial and all public hospitals in NSW

^e^As observed in No-CAUTI trial data

^f^Modelled estimates of averages for twelve month post-intervention period

All Costs inflated to 2019-2020 prices

#### Offset treatment costs

This study also includes offset service and treatment costs including catheterisations, urine tests and antibiotics [27, 43, 44]. The cost of each catheterisation equals that of a catheter pack, $8.14. The cost of nurse labour has been excluded, as administrating nurses would be hospitals employees, requiring wage payment without extra budget impact. Urine specimen culture testing was valued at $35.23 (MBS 69333, 73930) [45]. The average cost for one course of antibiotics was estimated at $19.48 (Additional file 1: Trial Antibiotics) based on costs from the Pharmaceutical Benefits Scheme (PBS) website [46]. These costs, along with modelling parameters are presented in Table 2.

#### Sensitivity scenarios

Sensitivity scenarios adjusted parameters and costs that have significant uncertainty including the intervention costs and catheterisation rates. Results indicate the scope of possible budget impact outside the base case. Scenarios 1 and 2 consider possible changes in the intervention costs.

1. Intervention Cost increases by 10%
2. Compliance Audits & Point Prevalence Surveys conducted by casual registered nurses (Year 8 and above)

The increase of 10% for Scenario 1, is a subjective but possible difference in real budget expenditure. For Scenario 2, the employment of casual nursing staff is a possibility if permanent hospital nurses are not available.

The remaining scenarios are adjustments to the estimated annual intervention catheterisation rate:

3. Increases 10%
4. Increases linearly to reach the usual care catheterisation rate by year 6

For Scenario 3, the 10% increase has been chosen subjectively, based on the assumption that over the five-year forecast, the average catheterisation rate could be significantly higher than described in the base case, but without equalling the usual care rate. Scenario 4 assumes that after Year 1, the catheterisation rate could trend back linearly to that of usual care. Such a gradual decay in No-CAUTI’s effect, may occur with decreasing compliance with No-CAUTI practice. Note that changes to catheterisation rates will also drive increases in urine testing and antibiotics administrations with associated cost increases.

#### Subgroup analysis – Metropolitan and Non-Metropolitan hospitals

Modelling was conducted for Metropolitan and Non-Metropolitan hospitals using point prevalence catheterisation, urine testing and antibiotics rates from the trial [13] (Additional file 1: Model Metro; Model non-Metro). Metropolitan and Non-Metropolitan hospitals were identified with reference to a NSW government information [47].

Hospital separations were allocated based on the sub-groups’ respective total bed numbers. Total intervention costs were allocated between sub-groups based on their respective numbers of planned workshops. In Year 1 Metropolitan hospitals are planned to receive 73% of workshops and in Years 2 to 5, 56% [13, 48] (Additional file 1: Model Metro; Model Non-Metro)*.*

### Ethics and trial registration

The study was granted ethical approval by the Hunter New England Human Research Ethics Committee (Ref no. 16/02/17/4.09) and the Central Coast Human Research Ethics Committee (reference no 1016-097C). Individual patient consent was not required for the point prevalence data collection as all data collected was from already routinely collected inpatient clinical data and in line with routine quality control auditing. All clinical data collected during point prevalence was de-identified. This approach was approved by the abovementioned human research ethics committees, Hunter New England Human Research Ethics Committee and the Central Coast Human Research Ethics Committee.

The study was also registered retrospectively with the Australian New Zealand Clinical Trials Registry (ACTRN12617000090314; first registration 17/01/2017, retrospective) [32]. The first hospital enrolment was on15/11/2016, and the last on 8/12/2016. All methods were conducted in accordance with relevant guidelines (i.e National Statement on Ethical Conduct in Human Research (Australia); the Consolidated Health Economic Evaluation Reporting Standards (CHEERS); Consolidated Standards of Reporting Trials (CONSORT) guidelines; and the StaRI checklist).

## Results

### Base case

Table 3 compares the budget costs and relevant service outcomes – of a No-CAUTI roll-out versus usual care – for the five-year time horizon (2022-23 to 2026-27), across 180 NSW public hospitals for an estimated 6.0 million overnight separations. As previously mentioned, the intervention cost for the five years is estimated at a $1.5 million. Over the period, it is estimated that annual overnight separations in NSW public hospitals will increase from approximately 1.15 to 1.22 million [49, 50] (Additional file 1: NSW Overnight Stays Forecast), (Table 3).

**Table 3:** No-CAUTI Roll Out: budget impact, new south wales public hospitals, five annual operating periods

			**2022-23**	**2023-24**	**2024-25**	**2025-26**	**2026-27**	**Total**
Estimated Annual Overnight Separations^1^	a		1,150,639	1,170,433	1,190,227	1,210,021	1,229,815	**5,951,135**
**Intervention Scenario**								
Catheterised patients								
Rate	b		10.27%	10.27%	10.27%	10.27%	10.27%	
(n)	c	b*a	118,182	120,215	122,248	124,282	126,315	**611,242**
Catheterised patients receiving urine tests								
Rate	d		45.20%	45.20%	45.20%	45.20%	45.20%	
(n)	e	d*c	53,408	54,326	55,245	56,164	57,083	**276,226**
Catheterised patients receiving antibiotics								
Rate	f		7.80%	7.80%	7.80%	7.80%	7.80%	
(n)	g	f*c	9,168	9,326	9,483	9,641	9,799	**47,417**
**Catheter Related Treatment Costs**								
Catheterisations($)	h	c*$8.14	$961,576	$978,118	$994,659	$1,011,201	$1,027,743	**$4,973,297 **
Urine Tests ($)	i	e*$35.23	$1,881,582	$1,913,950	$1,946,318	$1,978,686	$2,011,054	**$9,731,590 **
Antibiotic administrations ($)	j	g*$19.48	$178,632	$181,705	$184,778	$187,851	$190,924	**$923,891 **
Total Intervention Cost ($)	k		$408,122	$269,625	$269,625	$269,625	$269,625	**$1,486,623 **
**Total Costs Intervention Scenario**			**$3,429,912 **	**$3,343,398 **	**$3,395,381 **	**$3,447,364 **	**$3,499,346 **	**$17,115,401 **
**Usual Care Scenario**								
Catheterised Patients								
Rate	l		11.97%	11.97%	11.97%	11.97%	11.97%	
(n)	m	l*a	137,700	140,069	142,438	144,806	147,175	**712,188**
Catheterised patients receiving urine tests								
Rate	n		43.50%	43.50%	43.50%	43.50%	43.50%	
(n)	o	n*m	59,851	60,881	61,910	62,940	63,970	**309,552**
Catheterised patients receiving antibiotics								
Rate	p		7.60%	7.60%	7.60%	7.60%	7.60%	
(n)	q	p*m	10,463	10,643	10,823	11,003	11,183	**54,117**
**Catheter Related Treatment Costs**								
Catheterisations($)	r	m*$8.14	$1,120,379	$1,139,653	$1,158,926	$1,178,200	$1,197,473	**$5,794,631 **
Urine Tests ($)	s	o*$35.23	$2,108,586	$2,144,859	$2,181,133	$2,217,406	$2,253,679	**$10,905,663 **
Antibiotic Administrations ($)	t	q*$19.48	$203,872	$207,379	$210,886	$214,393	$217,900	**$1,054,430 **
**Total Costs Usual Care Scenario**			**$3,432,837 **	**$3,491,891 **	**$3,550,945 **	**$3,609,999 **	**$3,669,052 **	**$17,754,724 **
**Usual Care - Intervention (Differences)**								
Catheterisation Rates (%)			-0.017	-0.017	-0.017	-0.017	-0.017	
Catheterisations (n)			-19,518	-19,853	-20,189	-20,525	-20,861	**-100,946**
Catheterised Patients Receiving urine tests			-6,443	-6,554	-6,665	-6,776	-6,887	**-33,325**
Catheterised Patients Receiving Antibiotics			-1,295	-1,318	-1,340	-1,362	-1,385	**-6,700**
***Costs***								
Catheterisations ($)			($158,803)	($161,535)	($164,267)	($166,999)	($169,730)	**($821,334)**
Urine Tests ($)			($227,005)	($230,910)	($234,815)	($238,720)	($242,625)	**($1,174,074)**
Antibiotic Administrations ($)			($25,239)	($25,674)	($26,108)	($26,542)	($26,976)	**($130,539)**
Intervention Cost ($)			$408,122	$269,625	$269,625	$269,625	$269,625	**$1,486,623 **
**Total Net Costs**			**($2,925)**	**($148,493)**	**($155,564)**	**($162,635)**	**($169,706)**	**($639,323)**

^1^ NSW Ministry of Health Annual Reports. Extrapolations based on linear projections. www.health.nsw.gov.au/AnnualReport.

Omitted hospitals: Justice Health (Psychiatric) and Sydney Children's Network

All costs and parameters derived from No-CAUTI trialAll costs inflation adjusted to AUD 2019-20 costs with health inflation indexes from AIHW. Health expenditure Australia 2017–18. Number 65 Cat. no. HWE 77. 2019

The BIA forecasts five-year reductions for relevant NSW public hospitals of 100,100 catheterisations; 33,300 urine tests; and 6,700 antibiotic administrations. A total estimated budget cost saving over the time horizon is an estimated $640,000 (Table 3).

### Sensitivity scenarios

**Fig. 1:**
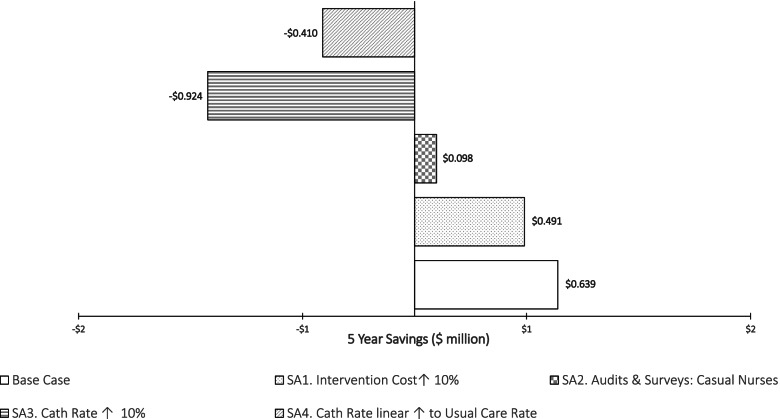
No-CAUTI Budget impact sensitivity scenarios: five years savings on usual care

Figure 1 shows five-year net cost results – No-CAUTI roll-out versus usual care – for all four sensitivity scenarios.

- Scenario 1 shifted the cost of the intervention upward by 10%. This would decrease net savings to $490,700 (23%).
- Scenario 2 costs a change to the intervention where audits and point prevalence surveys are performed by casual nurses, thereby increasing budget costs. This would significantly lower the five-year saving by 84.7% to $98,100.
- Scenario 3 considers a 10% increase in the estimated five-year catheterisation rate (10.27% to 11.3%) for the intervention. This would eliminate intervention savings and lead to a five-year cost of intervention of $923,600.
- Scenario 4 considers a linear increase in the intervention catheterisation rate – or a decay in the effect – back to the usual care catheterisation rate by year 6. This is predicted to generate a five-year budget cost of $410,300 (Additional file: Sensitivity Analyses).

The overall difference in cost/savings between the intervention and usual care is – given the scenarios conducted – most sensitive to changes in the expected catheterisation rate/s. This is partly because changes in catheterisations subsequent drive further changes and costs for urine tests and antibiotics.

### Subgroup analysis

Non-Metro hospitals showed a five-year saving of $1.2 million, while Metropolitan hospitals registered a loss of $539,000. This difference is mainly due to the greater effectiveness of the intervention in Non-Metropolitan hospitals. Tables can be viewed in Additional file 1: Table Metro and Table Non-Metro*.*

## Discussion

The authors have also conducted an in-trial cost-effectiveness analysis of the No-CAUTI intervention [51]. The results showed a positive incremental cost effectiveness for the intervention of $188 at 4 months post-intervention and $264 at 9 months post-intervention. These results indicate the expected cost efficiency for the intervention.

On its own, decision makers would find this economic analysis necessary but not sufficient for informed decision making. Missing in a pure economic analysis is an understanding of how No-CAUTI will impact on operational budgets. Questions that decision makers might pose are, ‘Will some existing healthcare stop as a consequence of the new model of care?’; ‘Is there a need for more/fewer staff?’; and, ‘Are additional capital purchases required?’. BIA is a means to leverage the economic analysis to provide more targeted information to decision makers and answer questions such as these. A BIA addresses the question of the financial cost, or affordability of intervention implementation. Consistently, the current study shows the projected cash outlays for the intervention in a budget period. Table 3 – a budget impact statement – details the costs of both intervention and usual care and the financial implications of offsets such as differences in expected costs for urine tests and antibiotics.

To the author’s best knowledge, this is the first Australian budget impact study (Additional file 1: Literature Review) of a nurse led catheter reduction program for hospitals aimed at reducing catheterisations and ultimately CAUTIs. This BIA study estimates that a five-year roll-out of No-CAUTI across 180 NSW hospitals, for 6 million overnight separations, will cost NSW Ministry of Health budgets approximately $1.5 million over five years. Urinary catheterisations would be expected to decrease on a no-intervention situation by 100,100 over 5 years and the intervention can save health budget funds from the first year of implementation. The net saving is estimated at approximately $3,000 in the first year and approximately $150,000 to $170,000 annually thereafter – a total of $640,000 over five annual operating periods (Table 3).

While the NSW health system may have alternative options, No-CAUTI shows improvements in budget costs and resource use, as compared to usual care. Any implementation of No-CAUTI would require measures to maintain practice adherence and effectiveness.

Scenario analyses showed that budget savings were most sensitive to adjustments in catheterisation rates. Increases in rates will result in loss of potential savings, requiring that a roll out make focused efforts to maintain intervention compliance. It is also noted that budget outcomes are different between metropolitan and non-metropolitan hospitals, with the later expected to accrue significant savings due to higher expected program effectiveness, while in metropolitan hospitals the intervention is expected to run at a cost. Hence a No-CAUTI roll out should focus on improved effectiveness in metropolitan settings.

Further budget efficiencies with the No-CAUTI program may be possible. For example, live streaming and on-line modules on the NSW government’s, Health, Education and Training (HETI) platform [52] could reduce the number of workshops required. A program of 20 workshops could be scheduled across a period of 6 months and be accessible to multiple sites concurrently. This would reduce the labour time and budget costs.

### Policy implications

Economic evaluations for operational health services should be accompanied by information that informs whether a new technology or model of care will be affordable. BIAs provide this information. Currently the guidelines for BIAs are mostly based on the ISPOR recommendations but these are international guidelines and not tailored to individual health systems. In Australia, and NSW specifically, work to customise BIA practice to local health systems is needed. The example of the BIA for No-CAUTI in this paper demonstrates how results and data from a cost effectiveness analysis can be leveraged to generate additional information for decision makers.

### Limitations

The study has limitations related to the No-CAUTI trial, from which it took most of its cost and parameter data. The trial was limited to only four hospitals, rather than a random robust sample of hospitals across NSW. The sample had no remote or very large Metropolitan hospitals. The point prevalence outcome measurement approach was not ideal as it failed to accurately summarise cumulative catheterisation rates for the entire 4- and 9-months post interventions periods. This budget analysis therefore required modelling to estimate annual post-intervention catheterisation rates and there is significant uncertainty for the pre-intervention catheterisation rate in its application for this study. It is noted that rollouts are likely to see variations in effect sizes and costs due to differences in quality control and patient mixes across hospitals.

## Conclusion

The findings demonstrate that an effective No-CAUTI implementation to reduce CAUTI cases and associated treatment costs is within the affordability of the Ministry of Health of NSW if it is can budget approximately $1.5 million for over five years. A roll-out at a state-wide level would be expected to save approximately between $640,000 in budget costs over five years while the base case is forecast to reduce catheterisations by over 100,100. Higher levels of intervention effectiveness would increase budget savings, freeing funds for other health care purposes. Rollouts are likely to see variations in effect sizes and costs due to differences in quality control and patient mixes among NSW public hospitals, but focused intervention management can bring implementation effectiveness within budgets.

## Supplementary Information

**Additional file 1.** Supporting Calculations referred to within the text.

## Data Availability

The data was sourced from the No-CAUTI trial (registration and ethics approvals details above) [13]. The data that supports the findings of this study are available from the authors, but restrictions apply to the availability of these data, which were used under license for the current study, and so are not publicly available. Data are however available from the authors upon reasonable request and with permission of co-author, Michelle Giles.

## Abbreviations

CAUTI: catheter-associated urinary tract infection
BIA: Budget Impact Analysis
NSW: New South Wales
ICU: intensive care unit
LHD: Local Health District
CNC3: Clinical Nurse Consultant Grade 3
RN8: Registered Nurse Year 8th year and thereafter
ACO6: Administrative and Clerical Officer Grade 6 Service
HETI: Health, Education and Training

## References

[1] Duckett SJ, Breadon P, Weidmann B, Nicola I (2014). Controlling costly care: a billiondollar hospital opportunity.

[2] Scott I (2014). Ten clinician-driven strategies for maximising value of Australian health care. Aust Health Rev.

[3] Productivity Commission. Efficiency in Health. Commission Research Paper. Canberra: Productivity Commission. 2015.

[4] Runciman WB, Hunt TD, Hannaford NA, Hibbert PD, Westbrook JI, Coiera EW, Day RO, Hindmarsh DM, McGlynn EA, Braithwaite J (2012). CareTrack: assessing the appropriateness of health care delivery in Australia. Med J Aust.

[5] Searles A, Gleeson M, Reeves P, Jorm C, Leeder S, Karnon J, Hiscock H, Skouteris H, Daly M. The Local Level Evaluation of Healthcare in Australia, Health Systems Improvement and Sustainability (HSIS) National Initiative. Newcastle: NSW Regional Health Partners; 2019.

[6] Australian Commission on Safety and Quality in Healthcare (2018). Hospital Acquired complication: Healthcare Associated Infections.

[7] Lo E, Nicolle LE, Coffin SE (2014). Strategies to prevent catheter-associated urinary tract infections in acute care hospitals: 2014 update. Infect Control Hosp Epidemiol.

[8] Mitchell BG, Ferguson JK, Anderson M, Sear J, Barnett A (2016). Length of stay and mortality associated with healthcare-associated urinary tract infections: a multi-state model. J Hosp Infect.

[9] Meddings J, Rogers MAM, Krein SL, Fakih MG, Olmsted RN, Saint S (2014). Reducing unnecessary urinary catheter use and other strategies to prevent catheter-associated urinary tract infection: an integrative review. BMJ Qual Saf.

[10] Saint S, Olmsted RN, Fakih MG, Kowalski CP, Watson SR, Sales AE, Krein SL (2009). Translating health care-associated urinary tract infection prevention research into practice via the bladder bundle. Jt Comm J Qual Patient Saf.

[11] Liu LF, Lee S, Chia PF, Chi SC, Yin YC (2012). Exploring the association between nurse workload and nurse-sensitive patient safety outcome indicators. J Nurs Res.

[12] Durant DJ (2017). Nurse-driven protocols and the prevention of catheter-associated urinary tract infections: A systematic review. Am J Infect Control.

[13] Giles M, Graham L, Ball J, King J, Watts W, Harris A, Oldmeadow C, Ling R, Paul M, O'Brien A (2020). Implementation of a multifaceted nurse-led intervention to reduce indwelling urinary catheter use in four Australian hospitals: A cluster controlled study. J Clin Nurs.

[14] Giles M, Watts W, O'Brien A, Berenger S, McNeil K, Bantawa K (2015). Does our bundle stack up! Innovative nurse-led changes for preventing catheter-associated urinary tract infection (CAUTI). Healthcare Infection.

[15] Giles M, Graham L, Ball J, Watts W, King J, Bantawa K, Paul M, Harris A, Paul O'Brien A, Parker V (2019). Variations in indwelling urinary catheter use in four Australian acute care hospitals. J Clin Nurs.

[16] Parker V, Giles M, Graham L, Suthers B, Watts W, O'Brien T, Searles A (2017). Avoiding inappropriate urinary catheter use and catheter-associated urinary tract infection (CAUTI): a pre-post control intervention study. BMC Health Serv Res.

[17] Sullivan SD, Mauskopf JA, Augustovski F, Jaime CJ, Lee KM, Minchin M, Orlewska E, Penna P, Rodriguez BJ-M, Shau W-Y (2014). Budget impact analysis-principles of good practice: report of the ISPOR 2012 Budget Impact Analysis Good Practice II Task Force. Value Health.

[18] Foroutan N, Tarride J, Feng X, Levine M (2018). A methodological review of national and transnational pharmaceutical budget impact analysis guidelines for new drug submissions. ClinicoEcon Outcomes Res.

[19] Trueman P, Drummond M, Hutton J (2001). Developing guidance for budget impact analysis. Pharmacoeconomics.

[20] Mauskopf JA, Sullivan SD, Annemans L, Caro J, Mullins CD, Nuijten M, Orlewska E, Watkins J, Trueman P (2007). Principles of Good Practice for Budget Impact Analysis: Report of the ISPOR Task Force on Good Research Practices—Budget Impact Analysis. Value Health.

[21] Faleiros DR, Alvares J, Almeida AM, de Araujo VE, Andrade EIG, Godman BB, Acurcio FA, Guerra Junior AA (2016). Budget impact analysis of medicines: updated systematic review and implications. Expert Rev Pharmacoecon Outcomes Res.

[22] Orlewska E, Gulacsi L (2009). Budget-impact analyses: a critical review of published studies. Pharmacoeconomics.

[23] Ghabri S, Autin E, Poullie AI, Josselin JM (2018). The French National Authority for Health (HAS) Guidelines for Conducting Budget Impact Analyses (BIA). Pharmacoeconomics.

[24] Health Information and Quality Authority. Guidelines for the Budget Impact Analysis of Health Technologies in Ireland. Cork: Health Information and Quality Authority; 2018.

[25] Palmer S, Dixon R (2019). Reducing catheter-associated urinary tract infections through best practice: Sherwood Forest Hospitals' experience. Br J Nurs.

[26] Pashnik B, Creta A, Alberti L (2017). Effectiveness of a Nurse-Led Initiative, Peer-to-Peer Teaching, on Organizational CAUTI Rates and Related Costs. J Nurs Care Qual.

[27] Apisarnthanarak A, Thongphubeth K, Sirinvaravong S, Kitkangvan D, Yuekyen C, Warachan B (2007). Effectiveness of multifaceted hospitalwide quality improvement programs featuring an intervention to remove unnecessary urinary catheters at a tertiary care center in Thailand. Infect Control Hosp Epidemiol.

[28] Anderson DJ, Miller BA, Chen LF, Adcock LH, Cook E, Cromer AL, Louis S, Thacker PA (2011). DJ S: The Network Approach for Prevention of Healthcare-Associated Infections: Long-Term Effect of Participation in the Duke Infection Control Outreach Network. Infect Control Hosp Epidemiol.

[29] Mitchell BG, Fasugba O, Cheng AC, Gregory V, Koerner J, Collignon P, Gardner A, N. G: Chlorhexidine versus saline in reducing the risk of catheter associated urinary tract infection: A cost-effectiveness analysis. Int J Nurs Stud 2019, 97:1-6. 10.1016/j.ijnurstu.2019.04.003 31129443

[30] Saint S, Kaufman SR (2005). Thompson M ea: A reminder reduces urinary catheterization in hospitalized patients. Jt Comm J.

[31] NSW Ministry of Health (Home Page). https://www.health.nsw.gov.au/Pages/default.aspx. Accessed 1 Aug 2020.

[32] Australian New Zealand Clinical Trials Registry. Trial Review https://www.anzctr.org.au/Trial/Registration/TrialReview.aspx? ACTRN=12617000090314%20ACTRN=12617000090314. Accessed 1 Aug 2020.

[33] Garattini L, van de Vooren K. Budget impact analysis in economic evaluation: a proposal for a clearer definition. Eur J Health Econ. 2011;12(6):499–502. 10.1007/s10198-011-0348-5 21874376

[34] NSW Ministry of Health. Health Stats NSW: Hospitalisations for All Causes. http://www.healthstats.nsw.gov.au/Indicator/bod_projhos/bod_hos_lhn. Accessed 20 Jan 2020.

[35] Australian Institute of Health and Welfare. Health Expenditure Australia 2017–18. Number 65 Cat. no. HWE 77, Canberra, ACT: Australian Institute of Health and Welfare. 2019.

[36] Australian Institute of Health and Welfare. Hospital Resources 2016–17: Australian Hospital Statistics. Canberra, ACT: Australian Institute of Health and Welfare. 2018.

[37] NSW Ministry of Health. Annual Reports. https://www.health.nsw.gov.au/annualreport/Pages/default.aspx. Accessed 26 May 2022.

[38] Microsoft Corporation. Microsoft Excel. Redmond: Microsoft Corporation; 2016.

[39] Australian Institute of Health and Welfare: Hospital resources 2018–19: Australian hospital statistics. Canberra: Australian Institute of Health and Welfare; 2020.

[40] Industrial Relations Commission of New South Wales. Public Health System Nurses' and Midwives' (State) Award 2018. Sydney: Industrial Relations Commission of New South Wales; 2018.

[41] NSW Government. Crown Employees (Public Sector - Salaries 2018) Award. Sydney: NSW Government; 2018.

[42] Western Sydney University. Office of Human Resources. On Costs https://www.westernsydney.edu.au/human_resources/ohr/on_costs. Accessed 30 July 2020.

[43] Clarke K, Tong D, Pan Y (2013). Reduction in catheter-associated urinary tract infections by bundling interventions. International J Qual Health Care.

[44] Sutherland T, Beloff J, McGrath C, Liu X, Pimentel MT, Kachalia A, Bates D, Urman RD (2015). A Single-Center Multidisciplinary Initiative to Reduce Catheter-Associated Urinary Tract Infection Rates: Quality and Financial Implications. Health Care Manag.

[45] Australian Government Department of Health. MBS Online. http://www.mbsonline.gov.au/internet/mbsonline/publishing.nsf/Content/Home. Accessed 23 Jan 2020.

[46] Australian Government Department of Health. Pharmaceutical Benefits Scheme. http://www.pbs.gov.au/pbs/home;jsessionid=b3d2i2oz7fx71bbi5o7mqkwd9. Accessed 23 Jan 2020.

[47] NSW Ministry of Health. Annual Report 2010-11 (Metropolitan Local Health Networks). https://www.health.nsw.gov.au/publications/Publications/Annual-Report-2010-11/13-NSW-HDs-Metro-Local-Health-Districts.pdf. Accessed 26 May 2022.

[48] NSW Ministry of Health. Local Health Districts and Specialty Networks. https://www.health.nsw.gov.au/lhd/Pages/default.aspx. Accessed 26 May 2022.

[49] Bureau of Health Information. Healthcare in Focus 2017: How Does NSW Compare?. Chatswood: Bureau of Health Information; 2018.

[50] Ministry of Health NSW. Annual Report 2017-18. Sydney: NSW Ministry of Health; 2018.

[51] Ling R, Giles M, Searles A. Administration of indwelling urinary catheters in four Australian Hospitals: cost-effectiveness analysis of a multifaceted nurse-led intervention. BMC Health Serv Res. 2021;21(897). 10.1186/s12913-021-06871-w PMC8408952 34465324

[52] NSW Ministry of Health. Health, Education and Training. https://www.heti.nsw.gov.au/. Accessed 31 May 2022.

